# Implications of autonomous shipping for maritime education and training: the cadet’s perspective

**DOI:** 10.1057/s41278-022-00217-x

**Published:** 2022-02-25

**Authors:** Krzysztof Bogusławski, Mateusz Gil, Jan Nasur, Krzysztof Wróbel

**Affiliations:** 1 Research Group on Maritime Transportation Risk and Safety, Gdynia Maritime University, Gdynia, Poland; 2 Marine Technology, Department of Mechanical Engineering, Aalto University, Espoo, Finland

**Keywords:** Maritime autonomous surface ships, Automation-induced underemployment, Maritime education, Education policy

## Abstract

The Industrial Revolution 4.0 has not left the transportation sector behind. All modes of transportation have, to some extent, already been affected, and maritime is the last to join them. Currently available technology makes autonomous merchant ships a possible alternative to conventional, manned vessels with seafarers. This upcoming shift requires the preparation of necessary policies, such as rethinking obsolete training curricula, in relation to a variety of aspects of the industry, including the future of seafaring as a profession. To formulate such policies, the views of professional seafarers and scholars are sometimes solicited, but the opinions of industry entrants are often neglected. However, the latter may also have some interesting views on the future of their profession, which may be relevant to policy-makers. The results of a worldwide survey, conducted using the Computer-Assisted Web Interviewing (CAWI) method, suggest that the future generation of seafarers fears automation less than their mentors. Although they expect their skills to be useful in automation-driven shipping, they also feel that their Maritime Education and Training institutions (MET) are not doing enough to prepare them for the challenges that the future may hold. This may be due to a lack or poor coverage of shipping autonomization issues in MET curricula, which was mentioned by as many as 41.9% of the respondents. This finding advocates for rethinking the curricula of METs and human resources management in the shipping industry of the future.

## Introduction

The maritime industry is about to undergo a technological revolution. For centuries, shipping has relied on manpower for setting sail, maintaining machinery, and plotting courses. However, this is soon going to change. With many modes of transportation already becoming automated or implementing autonomous systems (Goodall 2020; Outay et al. 2020), the maritime industry is lagging behind. However, it will eventually catch up—sooner rather than later.

Nowadays, shipping is an international business that employs some 1.6 million seafarers (UNCTAD 2019) and carries around 90% of global freight (Oksavik et al. 2020). However, it is often postulated that the safety and efficiency of the industry could be improved by removing humans from the direct loop (Wróbel et al. 2017; Hoem et al. 2019). Accordingly, automated ocean-going merchant vessels, also known as Maritime Autonomous Surface Ships (MASSs) (IMO MSC 2018) would navigate the sea either completely without human supervision or under control from a distant land-based facility, sometimes referred to as a shore control center (SCC) (Størkersen 2021; Ramos et al. 2019).

Depending on the degree to which such systems would be automated, human involvement in sea-going operations may be reduced from direct, manual control to remote supervision and control, or periodical condition-checking. In any case, the role of humans will decline: in some concepts, only a maintenance crew will be kept on board for a given period (Komianos 2018) instead of a full complement of crew. For others, all operations will be carried out remotely or autonomously with the use of an extensive network of sensors and communication links (IMO MSC 2018; Kobyliński 2018; Fan et al. 2020).

In the absence of legal coverage and the prevailing uncertainty towards current business and safety performance (Goerlandt 2020), it remains unclear *when* such a shift would occur or how it would result in technological advancement and social change. Nonetheless, experts agree that it eventually will (Kooij et al. 2019). This pursuit is driven by various factors, including cost reduction (Zghyer et al. 2019; Jo and Enrico 2020). Since labor costs on board can account for up to 36% of all costs (Kretschmann et al. 2017), the idea of autonomous (or unmanned) ships is tempting.

Making ships unmanned would result in leaving their crews stranded. The people normally needed to safely navigate and maintain ships might soon be deputized by computerized solutions. Moreover, after such replacement, a vessel might be supervised by other machines or by people who lack—at least at the start—familiarity with the maritime environment. Meanwhile, the educational model of seafarers and especially officers involves months—or even years—of practical training on board. This part of the professional education is compulsory in addition to even longer theoretical training at shore facilities (Maritime Education and Training (MET) institutions). These may also form a part of a higher education system in some countries. It is however a feature typically unknown to other transportation domains (except aviation), where vehicle *drivers* are not normally expected to pursue tertiary education. This provides a good opportunity to examine how young people and future seafarers, also referred to as *cadets* (Glen 2008), view the upcoming transformations in shipping and to their employment just before it becomes a reality. On the one hand, they are not yet qualified as ship *drivers* (officers) and therefore can hardly be called *experts*. On the other hand, they have decided to pursue their long-term (or even lifelong) career in this industry and study to do so. Thus, they are very likely to experience the changes that the industry will undergo within their own gainful employment. Therefore, they must be vitally interested in the future of their workplace. As shipping industry stakeholders and those who will shape its future, their opinions ought to be taken into consideration when developing international policies on maritime automation, education models, and employment. However, they rarely are—a gap our current research aims to bridge.

The issue of preparing the maritime workforce for the upcoming transformation has already been discussed by numerous authors. Sharma et al. (2019) carried out an expert study to verify the importance of seafarer competence standards to MASS. Fan et al. (2020) raised the point that training and education are factors affecting the operational risks of MASS. Janßen et al. (2021) argued that it is not only training, but also the experience of future remote operators that must be investigated. Finally, Nautilus Federation (2018) in their expert-based research concluded that training and reskilling is among the biggest obstacles for MASS adoption, although it was ranked as far less relevant than the reliability of communication links or legal issues. Training-related issues were also mentioned in other occasions, which indicates a research gap to be addressed—a gap between the future shape of the industry and the way people are trained to face it (Pietrzykowski and Hajduk 2019; Hogg and Ghosh 2016).

However, to the best of our knowledge, the attitudes and viewpoints of the most relevant stakeholders of METs, the students, have not been investigated to date. Some research concerning mariners’ attitude towards MASS has been conducted in recent years, but mainly focused on the opinion of experienced seafarers (Nautilus Federation 2018; Kim and Mallam 2020) or scholars/practitioners (Mallam et al. 2020). However, it is the entrants into the maritime industry who will be the most affected by the workforce-related implications of MASS. Given the technology preparedness level, the timeline of its development, the transition period, and the more than two decades life expectancy of manned vessels launched nowadays, a large portion of experienced and currently active seafarers will long be retired before a significant fraction of vessels become autonomous. However, those who are currently cadets may experience these changes and be directly affected by them. On the other hand, some of today’s entrants will at some point quit seafaring and apply their skills to land-based management positions. Nevertheless, they will still shape the future of this industry, its policies, and practices in relation to both technology and society. Therefore, investigating their mindset at the early stages of their career may provide an interesting insight into the future of Shipping 4.0.

To this end, the objective of the present study is to identify the attitude of future seafarers towards autonomous shipping and its implications for the job market. Specific factors to be addressed include:

1. Identifying the level of preparedness of METs for the challenges of autonomization
2. Investigating whether cadets fear being displaced from seafaring jobs by autonomous control
3. Estimating when such disruptive changes may occur
4. Determining whether cadets fear that their skills could become obsolete in an automation-dense maritime industry

The results were obtained through the application of the Computer-Assisted Web Interviewing (CAWI) method. The results indicate that cadets fear being pushed out of the job market more than professionals do. However, they expect that their learnt skills will help them find a job in a different branch of the maritime industry.

The remainder of the paper is organized as follows: firstly, the methods are introduced along with the materials, i.e., the demographic data of the respondents. Then, the results of the survey are presented and discussed. Based on these, policy implications are formulated. The final sections outline the uncertainties associated with the study and conclude the paper.

## Methods and materials

**Fig. 1:**
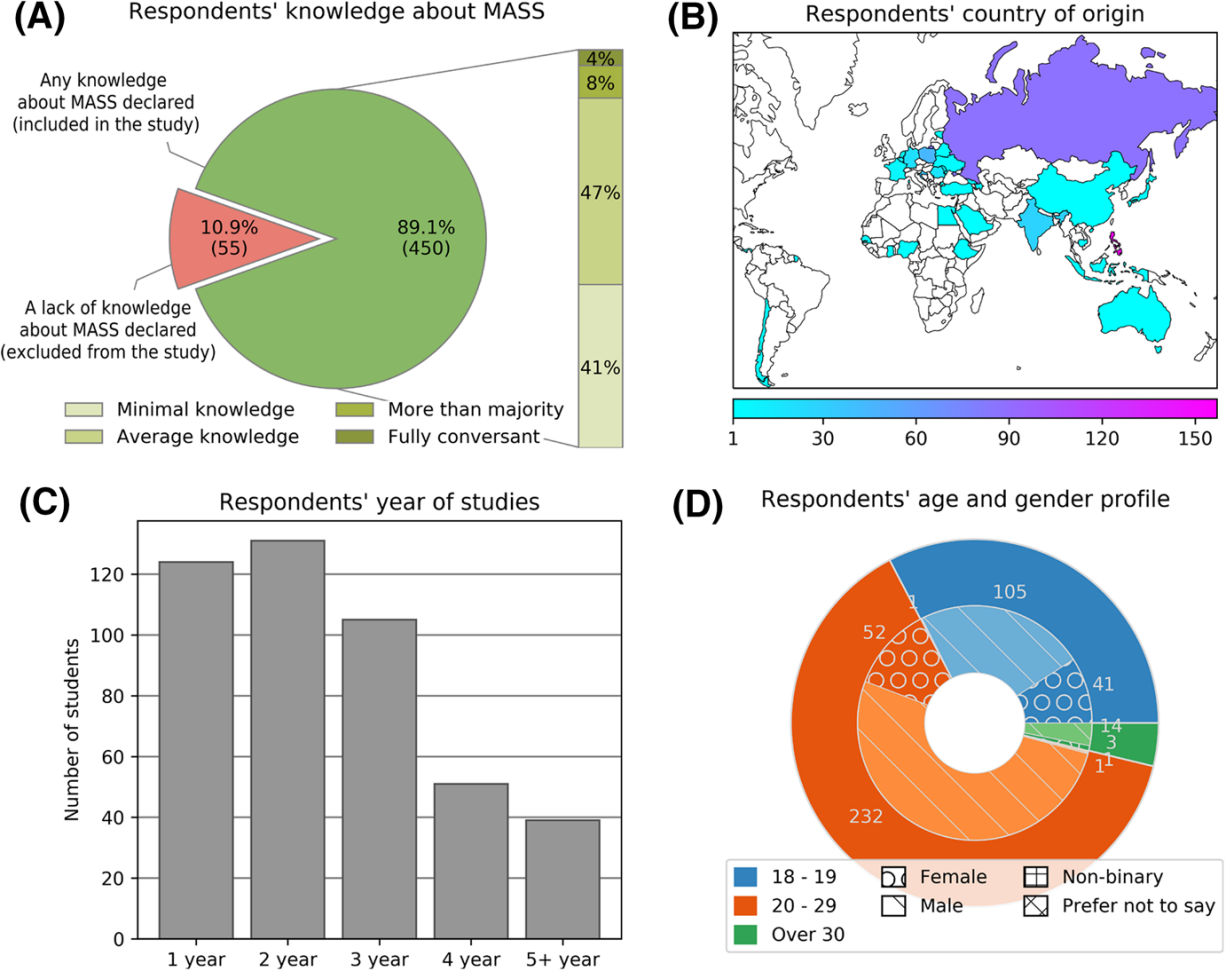
Demographic data of cadets elicited

As mentioned above, the entrants’ opinions were elicited through the Computer-Assisted Web Interview (CAWI) technique. This method was chosen based on the facts that (1) it enables worldwide elicitation of opinions and (2) other methods could hardly be used during the lockdown related to the COVID-19 pandemic. To this end, MET officials—members of the International Association of Maritime Universities (IAMU)—were requested to distribute the questionnaire among their students. Cadets were requested to share their views on MASS by filling out an online form. The survey was conducted in the spring of 2020. Eventually, cadets from 16 METs, located in nine UN-defined (UN, 1996) regions, participated. Of 505 returned and properly filled-in questionnaires, 450 met our eligibility criteria (89.1% acceptance rate). These included, above all, (1) self-declaring that issues related to maritime autonomous systems were *known* to the individual, and (2) not providing irrational answers (i.e., random strings, of which no such cases were found within the sample). It is noteworthy that the topic of MASS and intelligent shipping was unknown to some 10.9% of participants (Fig. 1A).

The design of the study allowed the inclusion of students from different departments, majors, universities, regions, and even with various levels of knowledge of MASS and intelligent shipping. The most prominent groups were those studying to work either on deck (future navigators) or in the engine department (future engineers and electrotechnical officers). However, maritime administration and logistics formed a notable group of 103 persons. Such an approach allowed further cross-sectional analysis, as well as drawing far-reaching implications depicting attitudinal differences among certain professions, both sea-going and shore-based, as presented in Sect. 3. On the other hand, it also burdened the study with some limitations, discussed in Sect. 5. Details of the demographic data of the students are shown in Fig. 1. Figure 1A shows a breakdown of the respondents by their self-declared level of familiarity with MASS. Note that almost 90% of respondents declared some degree of knowledge on MASS; of them, 47% had average knowledge and 12% above that. Figure 1B depicts their breakdown by country of origin, with the vast majority being from the Philippines (157), Russia (87), Poland (45), and Croatia (38). Figure 1C and D present the breakdown by study time, as well as the age and gender of the respondents. Almost 80% of the survey participants were studying in the first cycle (up to three years). For this reason, the youngest age group (18–19) represented a significant part of the whole, amounting to 33%. The remaining 63% belonged to the largest age group (20–29), as well as the oldest respondents (4%). Concerning gender diversity, males were the majority (78%), while 21% of all respondents were female. Note that the fraction of females in each age group was quite similar, varying from 17% to 18% in older groups to 28% in the youngest one.

## Results

The survey allowed us to collect the opinions of entrants on 29 different aspects of prospective MASS and of working in the era of intelligent shipping. A few of them were selected for detailed analysis and are presented below. The data breakdowns depicted in the figures were prepared for various demographic aspects of the respondents. To maintain a meaningful presentation, a group is only depicted if it comprises at least five answers (more than 1% of *n* = 450).

### METs could prepare themselves better for implementation of MASS

**Fig. 2:**
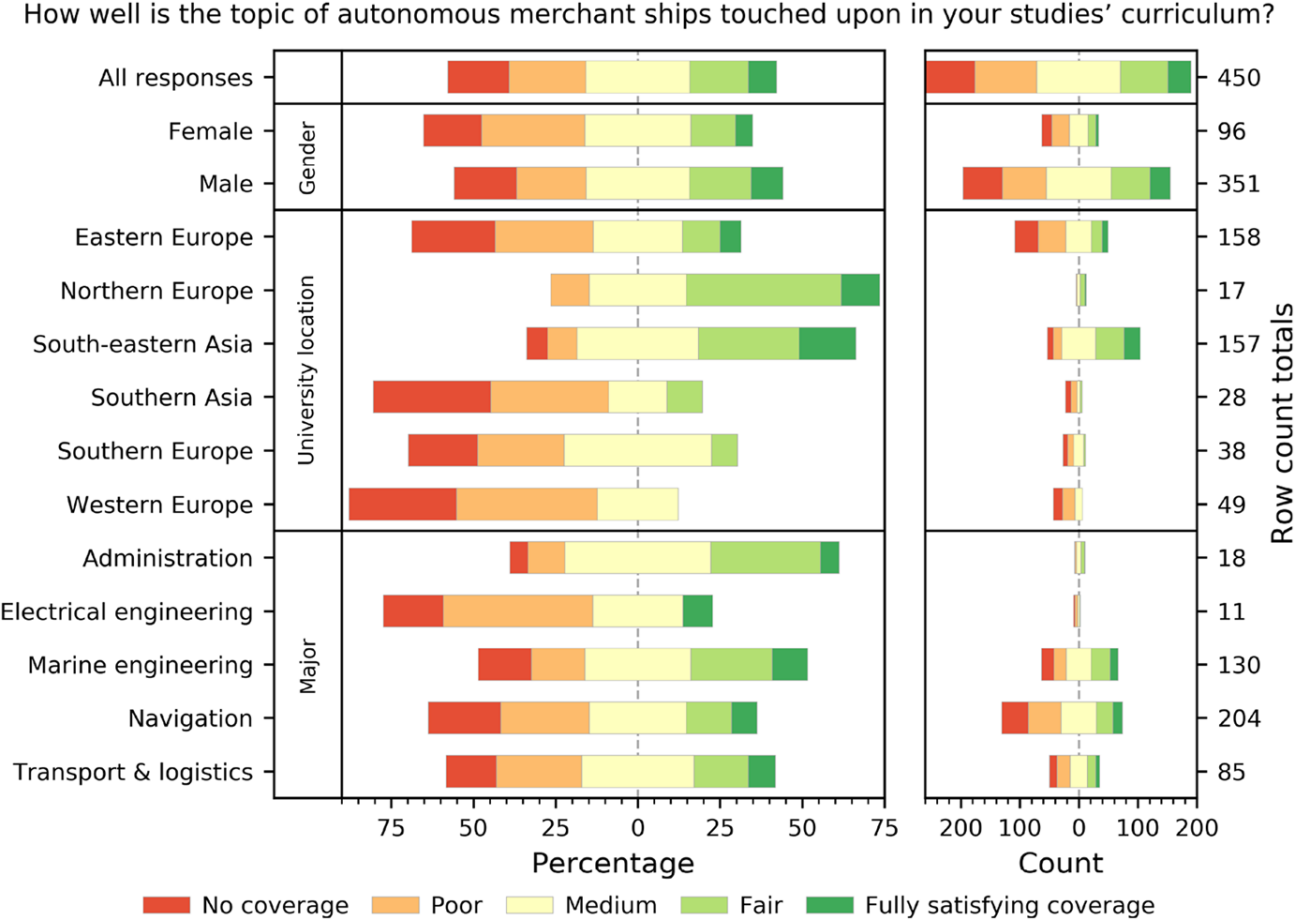
How cadets perceive the preparedness of METs for the upcoming implementation of MASS

Many cadets responded that issues related to autonomization of the shipping industry are not covered at all in their MET curricula, or are poorly covered (18.6% and 23.3% respectively, 41.9% in total; Fig. 2). Therefore, a significant part of the respondents declared they have only minimal knowledge related to this technology that has potential for disruptive change to the maritime industry. Only a quarter answered that such coverage is either *fair* or *fully satisfying* (26.4% altogether). It is noteworthy that the latter answers were more frequent among cadets studying in the Southeast Asia region (47.8%). This region is well known for supplying the global shipping industry with nationals who work as both ratings and for on-board management. However, the region is not normally associated with the development of highly innovative technical solutions for the industry. The fact that cadets claimed to have been familiar with the details of this prospective technology may indicate that the challenges (and opportunities) brought by it have been identified by the appropriate authorities. This might also indicate that, in light of being *seafarer suppliers* (Glen 2008) rather than *technology developers*, maritime industries in Southeast Asia^1^ decided that they must prepare their professionals for the upcoming shift rather than risk being left behind. Automation-induced unemployment would be devastating for communities that are strongly reliant on their sea-going members. On the other side of the spectrum were cadets from Western Europe. These evaluated the MASS coverage as either nonexistent or poor, medium at best. Some of the reasons for this may be (1) the fact that this topic is almost completely neglected by METs in these developed countries, or (2) the coverage itself is decent but students subjectively find it insufficient. The latter possibility may indicate that Western Europeans have higher expectations regarding training in innovative technologies and that METs fail to meet this demand for some reason. This in turn may indicate that, although Western and Northern Europe remain centers of R&D related to maritime autonomy, the training is lagging behind. This can be observed more in Western than Northern Europe.

### Cadets consider autonomization a threat to their potential workplace

**Fig. 3:**
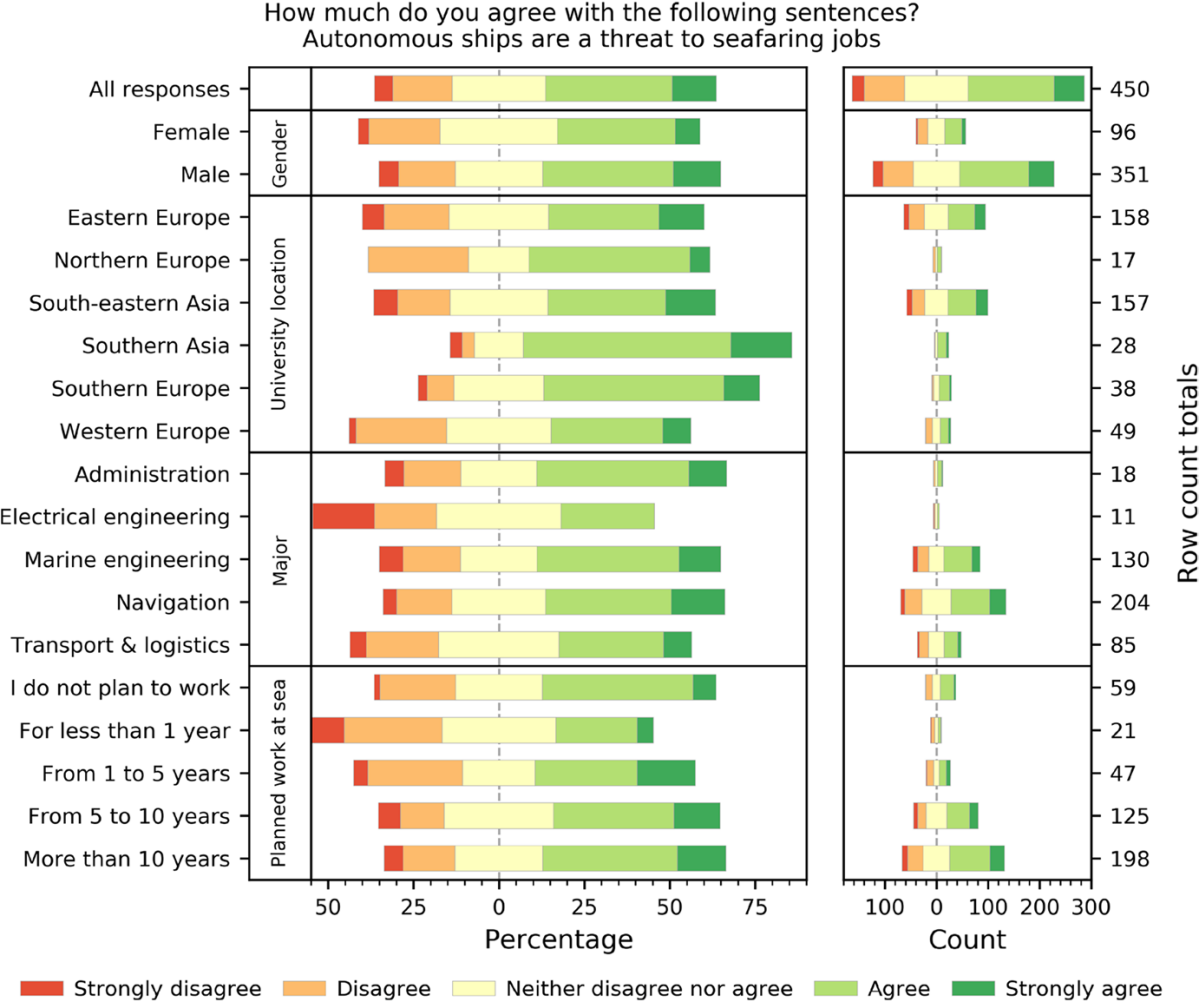
Cadets perceive automation as a threat to seafaring jobs

Since the knowledge of future maritime autonomous systems that cadets can gain from their training institutions is, to date, somewhat limited, it is of no surprise that they consider it a threat to seafaring jobs (50%; Fig. 3). The number is significantly lower than that reported among active professional seafarers, where it equaled 84% (Nautilus Federation 2018). It must be understood that the name by which MASS is sometimes referred to, i.e., *unmanned merchant vessels*, implies that there will be no one on board. Although the size of the global merchant fleet has been increasing in recent years (UNCTAD 2019), the introduction of autonomous vessels might result in the number of sea-going workplaces shrinking faster than the fleet’s growth. This effect can be, to some extent, compensated by the creation of shore-based jobs for seafarers in SCCs, as well as in other services. However, being shore-based, these jobs will not be in line with the “join the (merchant) navy, see the world” trademark used to attract young people to pursue a demanding, sea-going career. Whether fleet management centers or SCCs will be set up in countries rich in a maritime-experienced workforce or whether some other factors (i.e., lower cost) will prevail remains unknown at the moment.

Some fears and doubts about entrants’ future careers might arise also due to their short-term prognosis about MASS development and implementation.

### MASS will likely be implemented by 2040

**Fig. 4:**
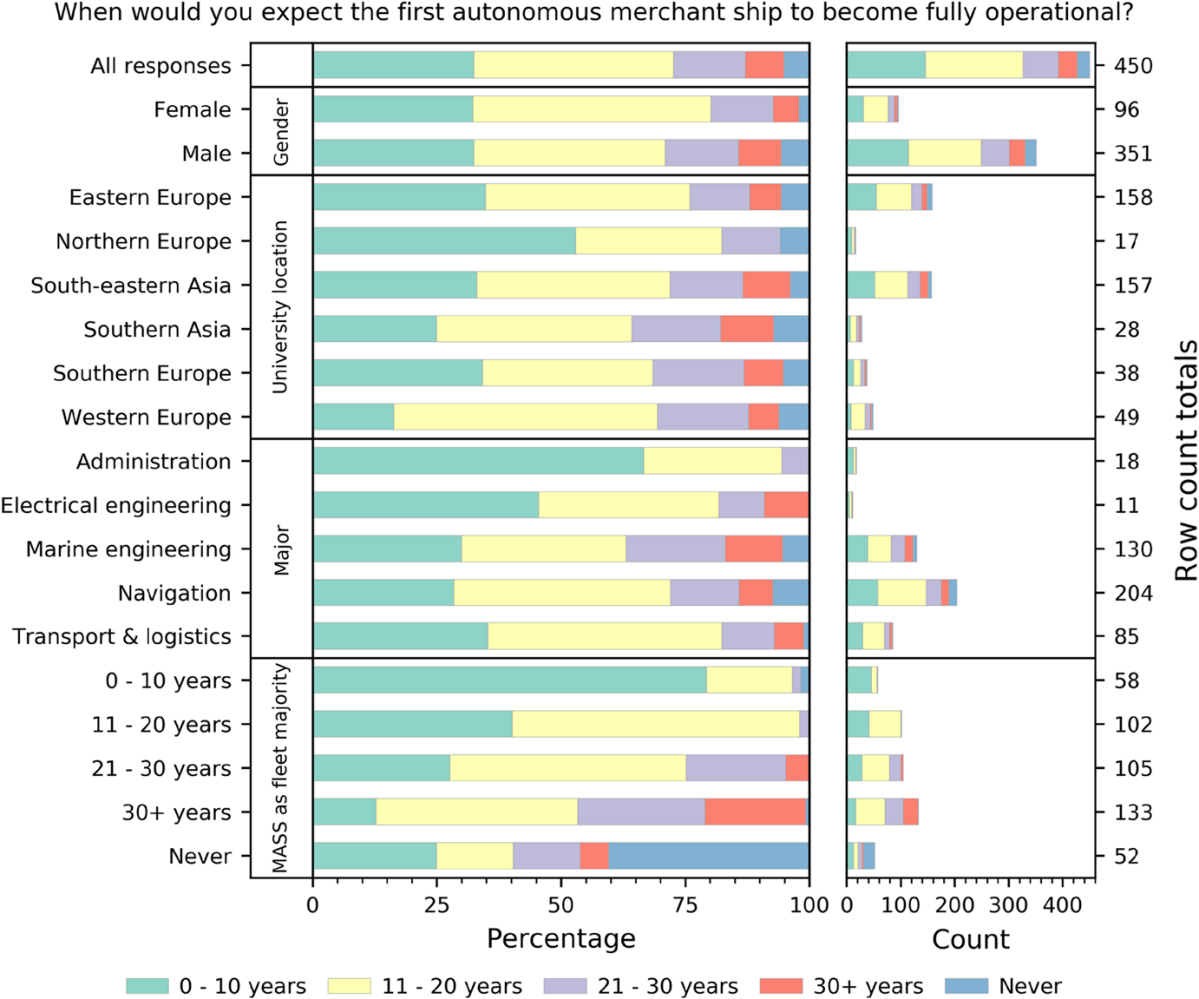
Expected timeline of MASS implementation

Most of the respondents (72.7%) agreed that, within 20 years, autonomous merchant ships would become fully operational (Fig. 4). Meanwhile, 32.4% predicted that this process will be finished by the next decade. Some more skepticism was expressed about the detailed time-span of MASS becoming the majority of the merchant fleet. The percentage of the *never* response equaled 11.6%. Relatively quick displacement (within a decade) of manned merchant ships was anticipated by a similar level (12.9%). Notably, the cadets studying sea-going majors, such as marine engineering or navigation, were more skeptical about the operational schedule of intelligent shipping. That may be due to the fact that, based on their (relatively limited) experience, they realize the challenges faced by the technology.

### Cadets’ skills will remain useful outside sea-going jobs

The vast majority (86.6%) of cadets admitted to considering the option of working ashore within the maritime sector. The answers were almost equally distributed among demographic groups, except for cadets majoring in electrical engineering, where the share was significantly smaller (63.6%).

**Fig. 5:**
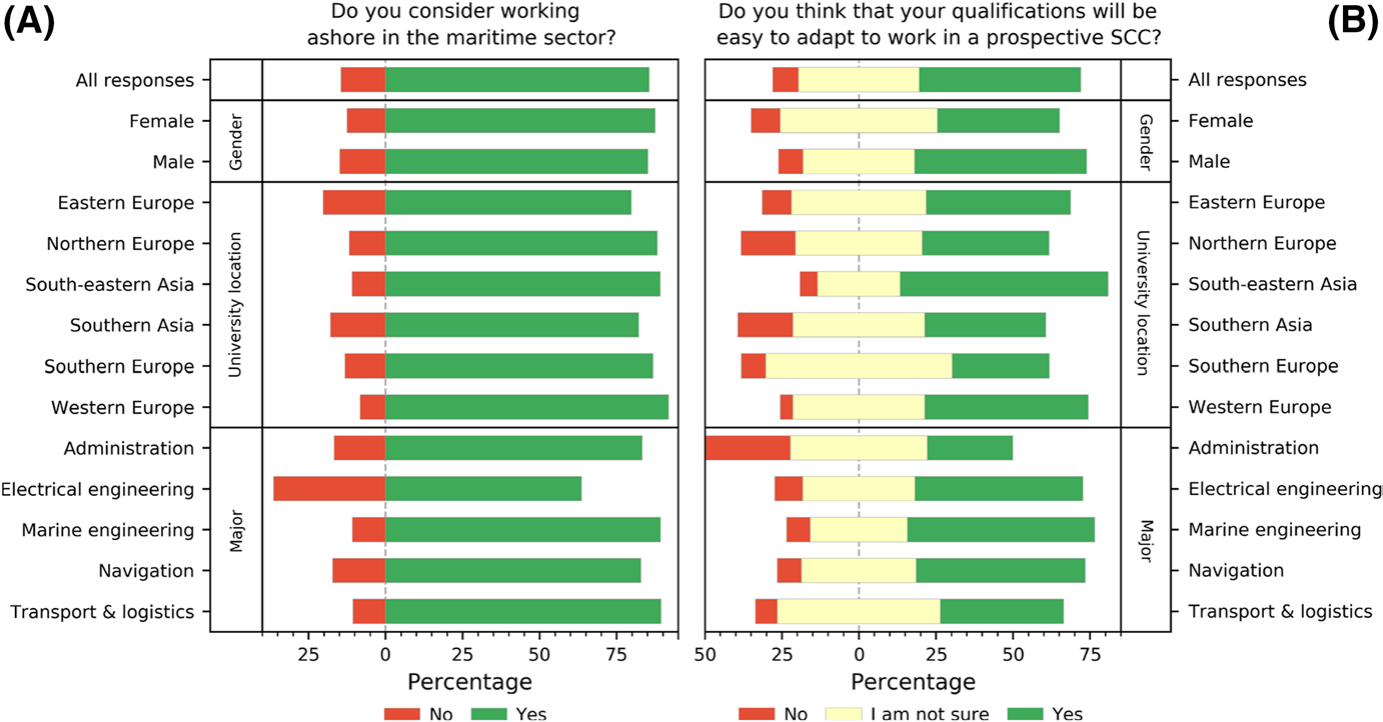
Cadets’ expectations towards potential land-based career and skills adaptability

Cadets were subsequently asked whether they thought that the skills they gain during the theoretical training and sea-going practice would be easily applicable to work within the SCC. The majority were optimistic in this respect (52.4%) or not sure (39.1%) (Fig. 5).

Notably, the most positive expectations towards potential land-based career and skills adaptability were again from cadets studying in Southeast Asia (where 67.5% thing that they might fit into these positions)—a good example of how implementing certain policies in education (even locally) can affect the attitudes of a social group. The least optimistic were cadets majoring in administration, perhaps because SCCs are normally described as facilities from where direct remote control over vessels and their equipment is carried out—a career profile not sought by future administrators.

**Fig. 6:**
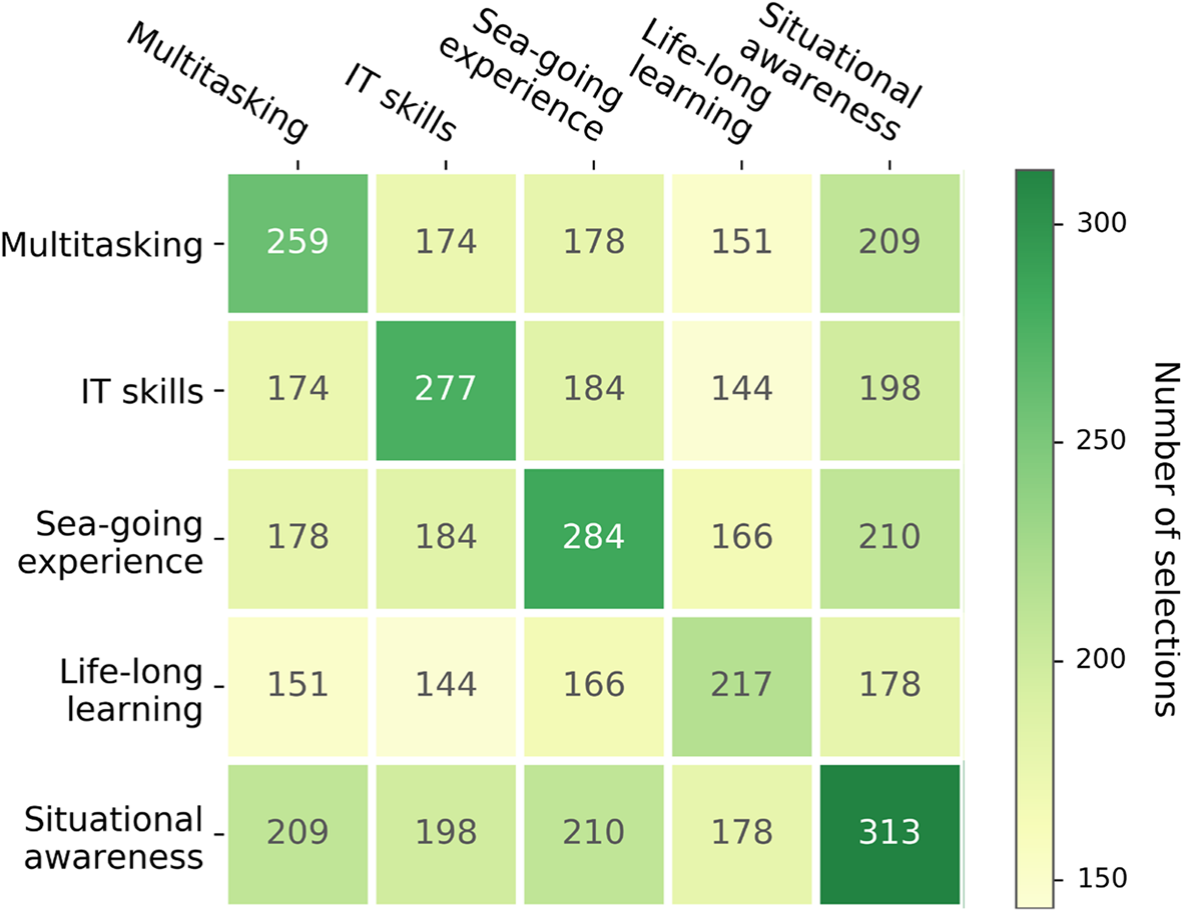
Cadets’ assessment of skills that will remain useful in automated shipping (the intersections of columns versus rows give the number of individuals selecting both respective answers, or one answer among the others for the main diagonal)

Although the discussed technology is still in its infancy, it is argued that a certain set of skills will be required to ensure the safety and efficiency of the system. These include remote situation awareness (Man et al. 2018), information technology proficiency (Oksavik et al. 2020), and the ability to share ship operator’s attention among different assets located around the world (Hogg and Ghosh 2016). The cadets were, in general, quite successful in identifying this set of skills, as presented in Fig. 6. This advocates that, at least in theory, they know what the future shipping industry will expect of them. Furthermore, those indicated skills overlap with those sought by the current seafaring industry (Kabir 2014). This may explain why the students of seafaring majors, such as navigation, marine engineering, and electrical engineering, were the most optimistic regarding the ability to adapt their qualifications to future work in prospective SCCs. They likely think of themselves as being well prepared for the challenges of the future of the industry, hoping that, even if their sea-going opportunities are reduced, they could find a shore-based job. By accepting the possibility of working ashore, the entrants appeared confident that they will hopefully get their bearings in a situation where they can no longer work at sea. In this way, one might infer that the sea-going careers of current cadets are unlikely to last their entire life—a trend that has been apparent for some time already (Obando-Rojas 1999; Caesar et al. 2015). Rather, it is an option chosen in certain circumstances. If these change, so can one’s career path.

## Discussion

In the following subsections, the results of this study are discussed, also leading to some policy recommendations. Last but not least, uncertainties of the research are presented and elaborated upon.

### Summary

The transition period from manned shipping as we know it today to an autonomous model is likely to take decades rather than years. Therefore, experienced seafarers may show a preference for the former. To these authors’ view, if there are too many seafarers in the market, those less experienced (and younger) may gradually be marginalized. If having extensive sea-going experience is not a prerequisite for a position within SCC, today’s entrants might still *sail* the ships remotely and exploit the advantageous office-like job. Given the results of this study, working ashore may be less of a problem for them than it may initially seem. Entrants do consider automation a threat to their workplace and thus acknowledge that they may eventually lose their jobs at sea. On the other hand, they do so less frequently than experienced professionals: 50% versus 84% as reported by Nautilus Federation (2018). Entrants do hope that the skills gained during theoretical and practical training will help them retain positions within the maritime industry, although not necessarily at sea.

Such optimism may be driven by at least two factors. Firstly, it may stem from a disbelief in the ability of the maritime industry to embrace autonomous control anytime soon. The industry is regarded as one that is slow to react to disruptive changes (Mallam et al. 2020; Ådland et al. 2004). Knowing this, cadets may believe that change, although negative in nature, will not occur soon enough, or be deep enough to affect their careers dramatically. Secondly, it may reflect the general view of Generation Z (to which the interviewed cadets belong) toward technology. It has been argued that this generation has been molded by technological development (Seemiller and Grace 2017). Students are generally more comfortable with the idea of autonomous transportation than the average population (Hudson et al. 2019). Although the actual rationale behind their answers in this research remains unknown, the students may simply share a positive attitude towards technical development in general. They perceive it as a tool to achieve their future goals rather than a phenomenon that can make them, as well as their skills, obsolete (Turner 2015). With this positive mindset, they are more confident that they could stand as competent crew members, able to meet the expectations of the new era. If not, then their skills will surely prove useful elsewhere, for example, in shore-based jobs related to the maritime industry, such as working in SCCs.

### Policy implications

With the above in mind, it can be summarized that, to manage the upcoming employment shift caused by increasing automation—up to autonomy in shipping, policy-makers should consider the following:

1. Increase the awareness of the future workforce with respect to their career prospects, by pursuing familiarization with novel, if not yet implemented, technologies—such as MASS. This could be accomplished by altering the curricula, or closer cooperation with industrial partners and R&D institutions. It is highly likely that the eventual implementation of autonomous merchant vessels will affect the sea-going labor market, just as the implementation of new technologies in ports changed their employment model (Haralambides 2017). Although it is estimated that some tens of thousands of sea-going positions remain open (Fernandez Gonzalez et al. 2014), it is currently difficult to predict exactly what the impact of MASS will be on sea-going employment. Entrants must be made aware that there exists a risk that their sea-going career path may close at some point, and that they will have to seek a different one. The latter may lie within land-based jobs (including remote control of autonomous ships), and almost 90% of entrants appear to accept this kind of job.
2. Redesign curricula and assist the future workforce in developing skills (including soft ones) useful outside sea-going operations rather than teaching hard technical subjects alone. Nowadays, the framework for training future navigators and engineers is based on an International Convention on Standards of Training, Certification, and Watchkeeping for Seafarers (STCW) and its related codes—legal documents that describe in detail the required technical skills. Due to this, certain METs may choose to only provide cadets with what is legally required for today’s shipping while neglecting a more general education. If this is the case, cadets (no matter how optimistic they are about their skills) may find themselves insufficiently prepared for the challenges of the market, threatened by increasing automation. For instance, IT skills may gradually become more important not only in shipping but also in a more technologically advanced society in general. It is argued that governance frameworks should be developed to help societies anticipate and shape the impact of emerging technologies (Baller et al. 2016). The same should be applied to maritime training, where this is currently done to a limited extent; indeed, 41% of entrants report having only minimal knowledge of the potentially disruptive technology of autonomous shipping.
3. Change training- and employment-related policies to accommodate the fact that the maritime industry may not be able to retain seafarers going forward. Depending on the rate, as well as on the long-term outcomes, of increasing autonomy in shipping, the current lack of experienced seafarers may deepen and must be addressed to secure the sustainability of the industry. On the other hand, such an effect may provide more arguments for developing MASS—to compensate for this lack of workforce. The causal relationship of these factors must be investigated further. As many as 42.9% of the interviewees expected to only work at sea for a limited amount of time (up to 10 years), while 13.1% declared that they do not consider sea-going work at all (note that the latter is lower than the share of non-sea-going majors in the research sample—22.9%). This further strengthens the argument for a need to provide more holistic training to the entrants. Once inside the maritime industry, cadets might gain more and more experience as they progress through their sea-going career, but eventually reach a decision that they wish to remain on land; thereby, they acquire skills that would primarily prove useful within this industry in various services, including prospective SCCs and similar facilities.

The above policy implications, for the sea-going labor market and the MET system, may be relevant to government bodies as well as to nongovernmental organizations addressing the educational aspects of the shipping industry. METs and their cadets may also be interested in learning the implications of this research, carried out for their respective purposes. More broadly, the issue of employing a skilled and experienced workforce in the maritime industry is of indisputable significance for global supply chains. Whether fully manned, remotely operated, or autonomous, sea-going merchant vessels will always require humans to run their operations and sustain international trade. The results of this study indicate that cadets are trained in the old-fashioned way (probably just to meet STCW requirements) that does not quite prepare them for the challenges of the future. Rethinking MET curricula is an obvious necessity going forward.

### Uncertainties and limitations

Like most other research, ours is not free of potential limitations that may affect the obtained results. Firstly, the overall response rate is not known due to the limitations of the survey platforms used. It can be assumed that the ongoing COVID-19 pandemic, forcing METs to switch to remote learning, may have limited the number of students to whom the survey was sent. How the pandemic, the related social isolation, and the economic slowdown may have affected the attitude of the cadets remains unclear. Secondly, while English is the dominant maritime language and should be known on at least a communicative level (Kartal et al. 2019), it was only a second language to most of the respondents. Misunderstandings of some of the questions could have occurred, which might have influenced the results. Lastly, the collected results on maritime students’ knowledge of MASS, as well as the topic coverage at their METs, may not fully reflect the actual situation, as such opinions are subjective in nature. This could be caused by merging answers from several different populations. In turn, this might have caused a cohort effect (Keyes et al. 2010), meaning that different groups of individuals could have had various opinions, depending on their social or cultural background. Drawing specific conclusions on, e.g., maritime education policies, should always take into account the specific needs of the cadets, their METs, and communities.

## Conclusions

The future of any society lies with its youth. This also applies to the seafaring community, without which global supply chains would collapse, that is now facing a disruptive change related to the expected arrival of autonomous control solutions. The opinions of industry entrants must, therefore, be acknowledged and observed to maintain sustainable social development. To this end, future seafarers fear that, at some point, they may no longer be needed and will need to find a different career path. This view is shared by fewer cadets than professional seafarers. The entrants do hope that, even if facing sea-going underemployment, they will find maritime-related jobs ashore, given that their skills will remain vital to the industry as a whole.

To face these issues, policy-makers are advised to (1) acknowledge that today’s cadets are not necessarily future captains, but perhaps also managers and remote pilots; (2) encourage their maritime workforce to also acquire other skills than those that are purely occupational; and (3) increase awareness of what the future of the industry may look like, and how their workers can fit into it.

One should, however, bear in mind that the conclusions and proposed policy-making recommendations presented herein apply to a very special industry, with many work-related peculiarities (i.e., acceptance of months-long isolation and related safety concerns). Moreover, the study itself is burdened with some uncertainties, the most important of which appears to be the subjectivity of the responses, provided by entry-level, to-be-seafarers, and other maritime professionals.

Nevertheless, it can be concluded that youngsters, although aware of the risks related to increasing automation, do acknowledge them and are rather optimistic with regard to their future in a highly computerized society.

## Footnotes

1 The Southeast Asia region includes a number of countries. In the context of the present study, most of the cadets from this region were of Filipino nationality, and this should be kept in mind when references to the “region,” or to SE Asia, are made.
